# Pentaploidization Enriches the Genetic Diversity of Wheat by Enhancing the Recombination of AB Genomes

**DOI:** 10.3389/fpls.2022.883868

**Published:** 2022-06-29

**Authors:** Fan Yang, Hongshen Wan, Jun Li, Qin Wang, Ning Yang, Xinguo Zhu, Zehou Liu, Yumin Yang, Wujun Ma, Xing Fan, Wuyun Yang, Yonghong Zhou

**Affiliations:** ^1^Triticeae Research Institute, Sichuan Agricultural University, Chengdu, China; ^2^Key Laboratory of Wheat Biology and Genetic Improvement in Southwestern China (Ministry of Agriculture and Rural Affairs of P.R.C.), Crop Research Institute, Sichuan Academy of Agricultural Sciences, Chengdu, China; ^3^Institute of Agricultural Resources and Environment, Sichuan Academy of Agricultural Sciences, Chengdu, China; ^4^Australia-China Joint Centre for Wheat Improvement, College of Science, Health, Engineering and Education, Murdoch University, Perth, WA, Australia

**Keywords:** pentaploid, chromosome recombination, linkage drag, genetic bottleneck, adaptive evolution

## Abstract

Allohexaploidization and continuous introgression play a key role in the origin and evolution of bread wheat. The genetic bottleneck of bread wheat resulting from limited germplasms involved in the origin and modern breeding may be compensated by gene flow from tetraploid wheat through introgressive hybridization. The inter-ploidy hybridization between hexaploid and tetraploid wheat generates pentaploid hybrids first, which absorbed genetic variations both from hexaploid and tetraploid wheat and have great potential for re-evolution and improvement in bread wheat. Therefore, understanding the effects of the pentaploid hybrid is of apparent significance in our understanding of the historic introgression and in informing breeding. In the current study, two sets of F_2_ populations of synthetic pentaploid wheat (SPW1 and SPW2) and synthetic hexaploid wheat (SHW1 and SHW2) were created to analyze differences in recombination frequency (RF) of AB genomes and distorted segregation of polymorphic SNP markers through SNP genotyping. Results suggested that (1) the recombination of AB genomes in the SPW populations was about 3- to 4-fold higher than that in the SHW populations, resulting from the significantly (*P* < 0.01) increased RF between adjacent and linked SNP loci, especially the variations that occurred in a pericentromeric region which would further enrich genetic diversity; (2) the crosses of hexaploid × tetraploid wheat could be an efficient way to produce pentaploid derivatives than the crosses of tetraploid × hexaploid wheat according to the higher germination rate found in the former crosses; (3) the high proportion of distorted segregation loci that skewed in favor of the female parent genotype/allele in the SPW populations might associate with the fitness and survival of the offspring. Based on the presented data, we propose that pentaploid hybrids should increasingly be used in wheat breeding. In addition, the contribution of gene flow from tetraploid wheat to bread wheat mediated by pentaploid introgressive hybridization also was discussed in the re-evolution of bread wheat.

## Introduction

Introgressive hybridization is generally considered to be a widespread and important occurrence during the evolution of flowering plants, although some researchers have described introgression as “evolutionary noise” (Anamthawat-Jónsson, 2001; Martin et al., 2007). Some introgression may be extensive, enabling the introgressed gene to move across species boundaries, with hybrids serving as bridges to gene flow, and resulting in species colonizing new habitats or exhibiting increased fitness in their existing niche (Martin et al., 2007). Consequently, introgressed genes are not confined to local hybridization events, making them important for the adaptive evolution of natural populations (Anderson, 1949; Rieseberg et al., 2003; Martin et al., 2007; Antoniou et al., 2018). Moreover, agricultural breeding largely depends on genetic recombination *via* introgression to generate new lines with enhanced traits derived from the genetic material in the parental chromosomes. Elucidating the extent and consequence of introgression provides insights into the phenotypic and genotypic changes during speciation, but it is also useful for evaluating the effects of gene flow, which may influence breeding applications.

Since introgressive hybridization requires the integration of maternal and paternal DNA, it depends on meiotic recombination, which facilitates the pairing of homologous chromosomes and their subsequent segregation in hybrids (Stapley et al., 2017). The evolutionary factors involved in the establishment of hybrids under natural conditions are considered to depend, at least partly, on the parental origin of particular genomic sequences involved in recombination (Soltis and Soltis, 2000; Leitch and Leitch, 2008). Moreover, the genetic variations from diploid relatives can enhance the evolutionary potential of species in the ever-changing environment (Leitch and Leitch, 2008). Empirical investigations revealed that the role of maternal and paternal chromosomes differs among chromosomes in flowering plants, with maternal rather than paternal chromosomes usually providing the genetic material that increases the fitness (e.g., endosperm development, seed production, germination, reproductive success, and survival) of the hybrid offspring (Bushell et al., 2003; Burgess and Husband, 2004; Kinoshita, 2008; Leitch and Leitch, 2008; Hernández et al., 2020). Inter-ploidy crosses with karyotypic imbalances also suggest that an excess of maternal genomic content (relative to the paternal genomic content) is important for seed development (Grossniklaus et al., 2001; Weiss et al., 2013). Recent studies demonstrated that manipulating the maternal genotype by genomic imprinting may result in offspring exhibiting the maternal phenotype (Iwasaki et al., 2019; Hernández et al., 2020). Although these studies clarified the maternal effects on the establishment of hybrids, changes in the frequency of maternal genotypes and the underlying molecular mechanism in hybrid populations remain unclear. Advances in DNA sequencing technologies and in the methods used to investigate variations in the frequency of maternal genotypes based on genome-wide variation data of populations may lead to the development of new strategies for precisely determining the role of maternal genotypes following hybridizations at the genome level.

Bread wheat (*Triticum aestivum* L., ABD genomes), one of the major sources of energy and protein in the modern human diet, was produced following two sequential hybridizations and domestication. The first hybridization (allotetraploidization) was between *Triticum urartu* (A) and an entity closely related to *Aegilops speltoides* (S), which resulted in tetraploid wild emmer (*Triticum dicoccoides*, AB genomes) approximately 0.5 to 3 million years ago (He et al., 2019). The second hybridization (allohexaploidization) was between the domesticated tetraploid wild emmer and *Aegilops tauschii* (D) nearly 8,000 to 10,000 years ago, which resulted in the formation of bread wheat (IWGSC, 2014; Zhang et al., 2014; He et al., 2019). However, wheat diversity has been limited by genetic bottlenecks caused by polyploidization, domestication, and modern breeding (Haudry et al., 2007; He et al., 2019). The bread wheat genome includes approximately 58% of the variants of wild emmer wheat (Luo et al., 2007), reflecting a large decrease in genetic diversity in the A and B genomes. A theoretical analysis (Dvorak et al., 2011) and empirical data for the chromosome pairing behavior of synthetic hexaploid wheat (Kerber, 1964), the molecular characterization of a diagnostic DNA marker in sympatric populations of wild emmer and hexaploid wheat (Dvorak et al., 2006), and exome sequencing results for hexaploid and tetraploid wheat (He et al., 2019) indicated that the genetic bottleneck of bread wheat may be compensated by the composite gene flow from tetraploid wheat (Zhou et al., 2020; Gaurav et al., 2021). The worldwide distribution of bread wheat implies that it must have some adaptive or selective advantages over its ancestral progenitor at the site of origin. These advantages may be not only from the addition of D genomes of *Ae. tauschii* species but also derived from the introgressive hybridization between hexaploid and tetraploid wheat. However, the process of introgressive hybridization through pentaploid hybrids during the evolution of hexaploid wheat remains uncharacterized since most previous studies regarding the genetic diversity of bread wheat largely focused on the long-term evolution of polyploids following the initial polyploidization events. Thus, the effects of pentaploid introgression during wheat breeding and evolution have yet to be characterized.

**FIGURE 1:**
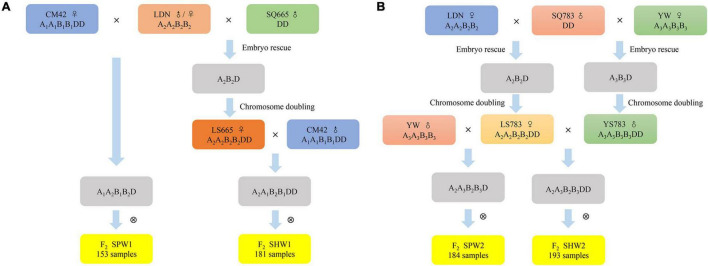
Procedures followed to create mapping populations. **(A)** CM42 and LS665 were used as the maternal donor to generate SPW1 and SHW1 populations, respectively. **(B)** LS783 was used as the maternal donor to generate SPW2 and SHW2 populations.

In this study, two sets of F_2_ populations of synthetic pentaploid wheat (SPW1 and SPW2) and synthetic hexaploid wheat (SHW1 and SHW2) were created to assess the recombination diversity of A and B genomes in the pentaploid and hexaploid genetic background and to reveal the role of interspecific introgression through pentaploid hybrids (Figure 1). The differences in reciprocal crosses, genetic recombination frequency (RF), and distorted segregation rate were analyzed between SPW and SHW populations. The results revealed that the crosses of hexaploid × tetraploid wheat showed a high proportion of germination rate than the crosses of tetraploid × hexaploid wheat; the genetic recombination of AB genomes was significantly (*P* < 0.01) increased in the SPW populations compared to the SHW populations; a high proportion of distorted segregation loci that skewed in favor of the female parent genotype/allele was found in the SPW populations compared to the SHW populations. During the introgressive hybridization between hexaploid and tetraploid wheat, the pentaploid hybrids might enrich the genetic diversity of bread wheat or durum wheat, generate numerous genetic variations, eliminate linkage drag and deleterious genotypes, and break the genetic bottleneck of contemporary wheat.

## Materials and Methods

### Plant Materials and Population Design

One SHW-derived hexaploid wheat, Chuanmai42 (CM42, A_1_A_1_B_1_B_1_DD), two tetraploid wheat, Langdon (*Triticum turgidum* conv. *durum*, LDN, A_2_A_2_B_2_B_2_) and Yuanwang (*T. turgidum* conv. *turgidum*, YW, A_3_A_3_B_3_B_3_), and two diploid *Ae. tauschii* accessions, SQ665 and SQ783, were used in this study. CM42 was released by the Sichuan Provincial Government in 2003 and remains widely grown in the southwestern region (Yang et al., 2009; Li et al., 2018; Aberkane et al., 2020). LDN was kept in Triticeae Research Institute, Sichuan Agricultural University. YW was collected by Dr. Wuyun Yang, Sichuan Academy of Agricultural Sciences (SAAS), China. SQ665 and SQ783 were provided by Dr. A. Mujeeb-Kazi, International Maize and Wheat Improvement Center (CIMMYT), Mexico, in 1995. High-ploid materials were employed as the female parent to produce hybrids.

Two tetraploid wheat and two diploid *Ae. tauschii* accessions were served as the female parents and male parents, respectively, to synthetic hexaploid wheat in 2011 (Figure 1). According to the works conducted by Zhu (2017) and Wan et al. (2021), three newly SHWs, LS665 (A_2_A_2_B_2_B_2_DD) derived from LDN × SQ665, LS783 (A_2_A_2_B_2_B_2_DD) derived from LDN × SQ783, and YS783 (A_3_A_3_B_3_B_3_DD) derived from YW × SQ783, were obtained with all ABD chromosomes validated by the non-denaturing fluorescence *in situ* hybridization (ND-FISH) (Cuadrado and Jouve, 2010; Tang et al., 2014; Fu et al., 2015). Hexaploid wheat, CM42, LS665, LS783, and YS783, and tetraploid wheat, LDN and YW, were used to construct pentaploid and hexaploid hybrids in 2015 (Figure 1).

The first SPW-derived F_2_ population (SPW1, 153 individuals) was produced by crossing CM42 with LDN (A_1_A_1_B_1_B_1_DD × A_2_A_2_B_2_B_2_) followed by selfing. The first SHW-derived population (SHW1, 181 individuals) was generated by crossing LS665 with CM42 (A_2_A_2_B_2_B_2_DD × A_1_A_1_B_1_B_1_DD) followed by selfing. The second SPW-derived population (SPW2, 184 individuals) was produced by a cross between LS783 and YW (A_2_A_2_B_2_B_2_DD × A_3_A_3_B_3_B_3_) followed by selfing. The second SHW-derived population (SHW2, 193 individuals) was generated from the hybridization between LS783 and YS783 (A_2_A_2_B_2_B_2_DD × A_3_A_3_B_3_B_3_DD) followed by selfing. Two sets of synthetic wheat populations, SPW1-SHW1 and SPW2-SHW2, were used to compare the genetic differences between pentaploids and hexaploids, including the distorted segregation, genetic maps, and recombination frequency.

In addition, reciprocal crosses were conducted using CM42 and LDN or LS783 and YW. The seed setting rate and germination rate of F_1_ pentaploid hybrids were statistical for analyzing the differences caused by the female parent with high-ploidy or low-ploidy level.

### Genotyping and Screen Polymorphism SNP Markers

Fresh leaves were collected from the four populations and their parents at the four-leaf stage. Genomic DNA was extracted from the leaves using the NuClean Plant Genomic DNA Kit (CWBio, Beijing, China). Genomic DNA samples (100 ng/μl) were sent to the China Golden Marker Biotechnology Co., Ltd. (Beijing, China) for genotyping using the 15K SNP chip array.

The values of quality control for the dish (DQC) and call rate (CR) for each SNP loci were calculated in SPWs or SHWs. Samples with DQC > 0.82 and CR > 91% were retained for genotyping. Genotyping analysis was conducted using the “apt-genotype-axiom,” “ps-metrics,” and “ps-classification” modules of the Affymetrix Axiom Analysis Suite software (version 4.0.1). SNP genotyping for SPW and SHW populations were separated regarding the relative fluorescent intensity of the A and B alleles to identify clusters when using “ps-classification” modules. For linkage analysis and map construction, the parents LDN, YW, CM42, LS665, LS783, and YS783 were all used to manually filter the polymorphic markers in AB genomes according to the following criteria: (1) SNP markers were only detected in the AB genomes; (2) SNP markers were polymorphic and homozygous between the parents of the corresponding population; (3) Each SNP markers in parents LDN and LS665 or parent CM42 should have the same genotypes both in the SPW1 and SHW1 populations; (4) Each SNP markers in parents LDN and LS783 or parents YW and YS783 should have the same genotypes both in the SPW2 and SHW2 populations; (5) SNP markers could identify homozygous and heterozygous genotypes in corresponding populations.

### Construction of the Genetic Linkage Map

The QTL IciMapping software (version 4.1.0) (Meng et al., 2015) was used to evaluate the RF and construct the genetic linkage map. The Kosambi mapping function was used to convert RF into centimorgan (cM) values (Vinod, 2011). To compare the RF between two genetic populations (SPW1-SHW1 or SPW2-SHW2), polymorphic markers were grouped and sorted according to their physical positions on the reference genome of Chinese Spring wheat (IWGSC version 1.0) (IWGSC, 2014) through the “By Anchor Order” and “Algorithm By Input” functions in QTL IciMapping (see the manual of QTL IciMapping). In addition, regarding the location change of SNP markers and the potential large genetic gap caused by the double crossover between adjacent and linked loci, the differences in genetic maps between SPW and SHW populations were validated through self-organization. By this method, the location of each SNP marker on each chromosome was sorted using the nearest neighbor (nnTwoOpt) algorithm (see the manual of QTL IciMapping).

Chi-square test analysis of all the polymorphic SNPs markers was performed to examine the deviations from the expected genotypic ratio 1:2:1 and allelic ratio 1:1 of CM42 and LDN for the F_2_ SPW1-SHW1 populations using Microsoft (MS) Excel 2019. The same analysis was also performed for the distorted segregation of LDN and YW in the F_2_ SPW2-SHW2 populations. The *t*-test of the IBM SPSS Statistics program (version 26) was used to analyze the genetic intervals or RF differences between adjacent and linked loci on each chromosome in the pentaploid and hexaploid populations. The markers potentially associated with genetic gaps in the hexaploid populations were removed before performing the *t*-test.

## Results

### Differences of Reciprocal Crosses in Seed set and Germination

Table 1 shows the differences in seed setting rate and germination rate in reciprocal crosses between several hexaploid and tetraploid wheat. The average seed setting rates were all about 50% in reciprocal crosses. The germination rates of hybrids were 52.35 and 54.77% in CM42/LDN and LS783/YW populations, respectively. However, the crosses of LDN/CM42 and YW/LS783 only showed an average 7.87 and 8.42% germination rate, respectively. A higher germination rate of hybrids was found while the hexaploid wheat (high-ploidy) was employed as a female parent in the reciprocal crosses.

**TABLE 1:** Statistical of seed setting rate and germination rate in reciprocal crosses.

Population	Florets pollinated	Seed set	Seed setting rate	Germination	Germination rate
CM42/LDN	612	277	45.26%	145	52.35%
LDN/CM42	582	285	48.97%	24	8.42%
LS783/YW	426	241	56.57%	132	54.77%
YW/LS783	674	343	50.89%	27	7.87%

*CM42: synthetic hexaploid wheat-derived variety Chunmai42; LDN: Triticum turgidum conv. durum, Langdon; LS783: LDN-Ae. tauschii accession SQ783 amphiploid line; YW: Triticum turgidum conv. turgidum, Yuanwang.*

### SNP Markers Distribution and Genetic Map Differences

A total of 13,199 SNPs were detected in four F_2_ populations, SPW1 (CM42/LDN), SHW1 (LS665/CM42), SPW2 (LS783/YW), and SHW2 (LS783/YS783), and 9,925 SNPs located on AB genomes through the wheat 15K SNP array. The number of SNPs anchored on AB chromosomes ranged from 463 on 6A and 997 on 3B (Supplementary Table 1). The polymorphic markers were manually filtered, leaving 1,650 SNPs (polymorphic rate: 16.62%) and 904 SNPs (polymorphic rate: 9.11%) in the SPW1-SHW1 and SPW2-SHW2 populations, respectively (Supplementary Table 1). In the SPW1-SHW1 populations, the number of polymorphic markers ranged from 27 on 6A to 229 on 7A and the ratio of polymorphic markers varied from 5.83% (6A) to 31.16% (7A). Some polymorphic loci were found located on the pericentromeric region in several chromosomes such as 3A, 7A, 3B, and 7B (Supplementary Figure 1). Some polymorphic loci were more concentrated on one region of the chromosome arm, such as the 500 to 720 Mb region on the long arm of 2A and 540 to 700 Mb region on the long arm of 5A. For the SPW2-SHW2 populations, the number of polymorphic markers ranged from 28 on 4B to 107 on 3B, and the ratio of polymorphic markers varied from 4.75% (4B) to 14.04% (6A). Chromosomes 1B, 2B, 3B, and 6B were found in some polymorphic SNPs loci located in the pericentromeric region. Chromosomes 1A, 2A, 5B, and 7B were found in many polymorphic loci clustered on the long arm. In conclusion, polymorphic markers generally increased abundance as the distance from the centromere increased. The ratio of polymorphic SNPs per chromosome in the SPW1-SHW1 populations was higher than that in the SPW2-SHW2 populations, while the polymorphic ratio of 6A in the SPW2-SHW2 populations increased by 8.21% compared with the SPW1-SHW1 population set. The average polymorphic ratio of A and B genomes showed no statistical difference in both two population sets.

**FIGURE 2:**
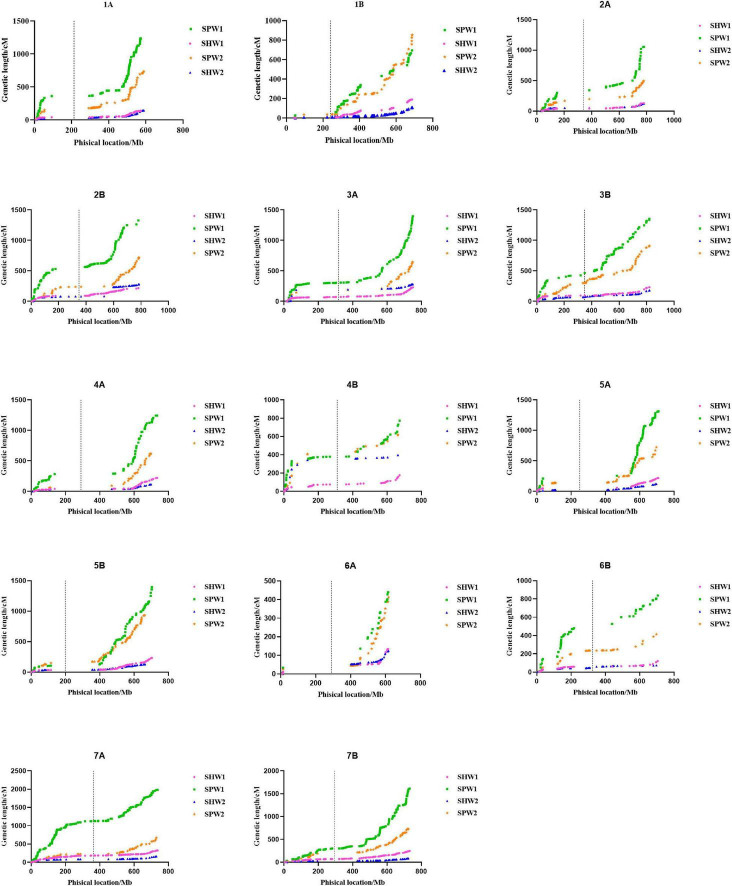
Comparison of the genetic maps of each chromosome between pentaploid and hexaploid populations. The position of SNP markers was aligned with the IWGSC genome (version 1.0). Dotted lines indicate the physical location of the centromere on each chromosome.

The first set of genetic maps was constructed according to the physical position of polymorphic SNP markers aligned with the reference genome CS (“By Anchor Order” and “Algorithm By Input” functions). Figure 2 presents the results of a comparative analysis of the genetic maps of SPW and SHW. The results showed that the length of the genetic maps of SPW1 (16,647.09 cM, 37 genetic gaps) and SPW2 (9,476.49 cM, 15 genetic gaps) were 4.92- and 3.07-fold longer than those of SHW1 (2,811.96 cM, 2 genetic gaps) and SHW2 (2,326.69 cM, 9 genetic gaps) (Supplementary Table 2), respectively. The genetic maps were also self-organized by sorting the location of SNP markers in each chromosome using the nnTwoOpt algorithm method. Compared with the first set of genetic maps, the self-organized genetic maps of SPW1 (7,388.85 cM) and SPW2 (4,005.36 cM) decreased more than half in length, whereas the SHW1 (2,396.91 cM) and SHW2 (1,931.90 cM) only decreased 415.05 and 394.79 cM (Supplementary Table 2), respectively. No genetic gap was found in SPW and SHW populations in the second set of genetic maps. Nevertheless, the self-organized genetic maps of SPW1 and SPW2 were more than three and twice as long as the SHW1 and SHW2, respectively. In general, the inter-ploidy hybridization between hexaploid wheat and tetraploid wheat greatly increased the genetic maps compared to the intra-ploidy hybridization of hexaploid wheat.

### AB Genomic Recombination in Pentaploid and Hexaploid Backgrounds

Regarding the huge difference in genetic maps between pentaploid- and hexaploid-derived populations, the genetic interval and recombination frequency between adjacent and linked loci on each chromosome were compared using the two-tailed paired-sample *t*-test (Supplementary Table 3). Compared with the SHW1 population, the pentaploid hybridization led to the average genetic interval on each chromosome increasing by 5.53 to 12.58 cM in the SPW1 population. The average genetic interval on each chromosome was also increased in the comparison between SPW2 and SHW2 populations, ranging from 4.43 cM (6A) to 10.76 cM (4A). These huge differences between pentaploid- and hexaploid-derived populations were attributed to the significantly (*P* < 0.01) increased genetic distance between two neighboring and linked SNP loci on each chromosome.

In addition, the inter-ploidy hybridization between hexaploid and tetraploid wheat led to the RF between adjacent and linked SNP loci increasing significantly (*P* < 0.01) (Supplementary Table 3). Consequently, the average RF in the SPW1 population increased by 4.55% on 1B to 10.67% on 6B compared to the SHW1 population and the average RF in the SPW2 population increased by 4.19% on 6A to 9.78% on 3A compared to the SHW2 population.

**FIGURE 3:**
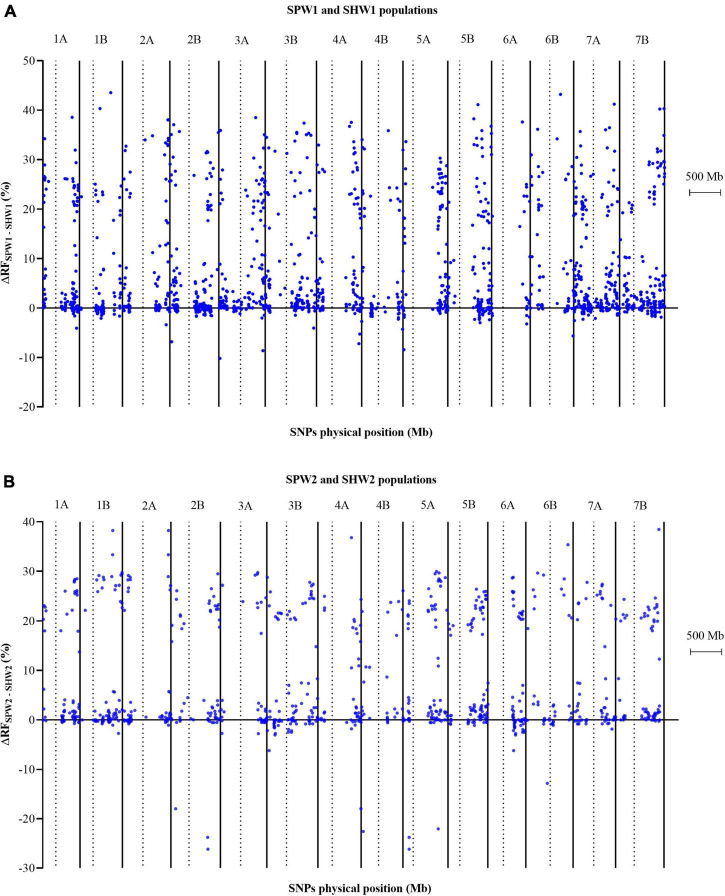
Comparison of the variation in recombination frequency between adjacent and linked SNP loci in pentaploid and hexaploid populations. Dotted lines indicate the physical location of the centromere on each chromosome. **(A)** SPW1 and SHW1 populations. **(B)** SPW2 and SHW2 populations.

In conclusion, the pentaploid hybridization could significantly enhance the RF between adjacent and linked SNP loci, resulting in significant increases in the genetic interval, as the genetic interval was converted by the RF between adjacent and linked SNP loci using the Kosambi model. Besides, the RF generally increased to varying degrees from the centromere to the telomere (Figure 3).

### Distorted Segregation Analysis

Chi-square testing revealed that 1,329 (80.55%) and 79 (4.79%) of 1,650 SNPs markers showed significant (*P* = 0.01) distorted segregation in the F_2_ SPW1 and SHW1 populations, respectively (Supplementary Table 4). In the SPW1 population, 1,171 (70.97%) distorted segregation loci (SDLs) skewed in favor of the female parent CM42 allele and the other 156 (9.45%) SDLs were biased toward the male parent LDN allele. These SDLs were distributed on all chromosomes, especially 2B and 7B. In the SHW1 population, 15 (0.91%) SDLs that skewed in favor of the female parent LS665 allele were distributed among six chromosomes as follows: 1A (5), 4A (2), 7A (2), 2B (1), 3B (3), and 6B (2). Sixty-four SDLs that skewed in favor of the male parent CM42 allele were distributed on chromosomes 2A (2), 5A (27), 6A (2), 1B (1), 3B (28), 4B (1), 5B (1), and 7B (2).

In the SPW2 and SHW2 populations, 577 (63.83%) and 58 (6.42%) of 904 SNPs markers showed significant (*P* = 0.01) distorted segregation, respectively (Supplementary Table 4). In the SPW2 population, 548 (60.62%) SDLs were skewed in favor of the female parent LS783 allele and distributed on all chromosomes. The other 29 (3.21%) SDLs skewed in favor of the male parent YW allele were distributed on all chromosomes except the group 2 and 6 chromosomes. However, 39 of 57 distorted segregation markers that skewed in favor of the female parent LS783 allele in the SHW2 population were all distributed on chromosomes 6B, followed by 17 on 4B and 1 on 7A. There was only 1 (0.21%) distorted marker biased toward the male parent YW allele and was distributed on chromosome 7B.

**FIGURE 4:**
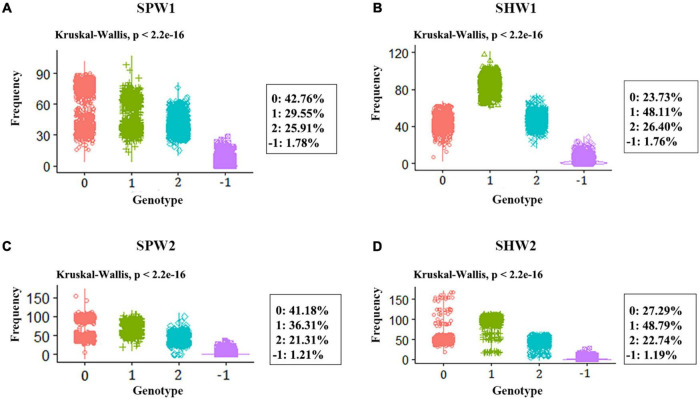
Statistical of genotype frequency in pentaploid and hexaploid populations. 0, genotype frequency of the female parent; 1, genotype frequency of the heterozygous; 2, genotype frequency of the male parent; -1, missing rate. **(A)** SPW1 population. **(B)** SHW1 population. **(C)** SPW2 population. **(D)** SHW2 population.

**FIGURE 5:**
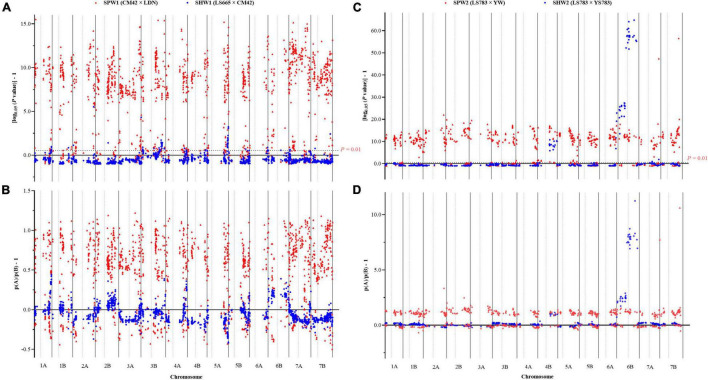
The distorted segregation **(A,C)** and parental gene bias **(B,D)** of SNP markers of AB genomes in SPW and SHW populations. *P*-value was calculated by Chi-square test with a theoretical genotype frequency of 1:2:1 (AA: AB: BB). If the [log0.05 (*P*-value) - 1] > 0 or 0.54 at a SNP site, significant distorted segregation was inferred at this locus at *P* = 0.05 or 0.01 level, respectively. *p* (A) means gene frequency of the female parent; *p* (B) means gene frequency of the male parent. If [*p* (A)/*p* (B)–1] > 0, the gene frequency of the female parent was higher than that of the male parent in the F_2_ recombination populations, and the genotype was biased toward the female parent at this locus.

In general, the genotype frequency of female parents (42.76 and 41.18% in SPW1 and SPW2, respectively) in each pentaploid derived population was much higher than the genotype frequency of heterozygous and male parent statistical from the genotyping results of all polymorphism SNP markers among all individuals (Figures 4A,C). In contrast, the genotype frequency of heterozygous (48.11 and 48.79% in SHW1 and SHW2, respectively) was much higher than the parental genotype frequencies (Figures 4B,D). The *P*-values of the Chi-square test against the expected genotypic segregation ratio of 1:2:1 in two F_2_ populations of synthetic pentaploid wheat was much higher than that of two F_2_ populations of synthetic hexaploid wheat (Figures 5A,C). The majority of the distorted segregation markers were skewed in favor of the female parent allele [*P* (A)] after the inter-ploidy hybridization between hexaploid wheat and tetraploid wheat, in which the B-subgenome showed a higher distorted segregation rate compared with the A-subgenome (Figures 5B,D). The high distorted segregation rate on chromosomes 4B and 6B in SHW2 that biased toward the female parent allele led to the skewed direction different from the SHW1 population, of which most SDLs were biased toward the male parent allele [*P* (B)].

## Discussion

### Pentaploid Hybridization Significantly Increases Homologous Recombination

Polyploid hybridization between tetraploid and diploid wheat gave rise to the allohexaploid wheat, which has become one of the major crops contributing to its genome plasticity (Dubcovsky and Dvorak, 2007; Liu et al., 2021). Since numerous desirable agronomic characteristics are contained in tetraploid wheat, many research focused on the introgression of beneficial genes and traits into hexaploid wheat (Xie and Nevo, 2008; Nevo et al., 2013; Peng et al., 2013; Nevo, 2014). The immediate outcome of the inter-ploidy hybridization between tetraploid and hexaploid wheat is pentaploid hybrids (Kihara, 1924). Therefore, studying the genetic dynamics and contributions of the pentaploid hybrids in the introgressive crossing between hexaploid and tetraploid wheat is of great importance in understanding the gene introgression between hexaploid and tetraploid wheat and the innovation and utilization of germplasm resources.

Previous studies indicated that the recombination frequency and recombination location could be manipulated through changing environmental conditions and silencing or knocking out the anti-crossover factors (Bennett and Rees, 1970; Boyko et al., 2007; Higgins et al., 2012; Andreuzza et al., 2015; Harper et al., 2018; Lloyd et al., 2018; Maagd et al., 2020). It was also suggested that an increase in recombination frequency might be associated with changes in the ploidy level (Desai et al., 2006; Leflon et al., 2010; Pecinka et al., 2011; Suay et al., 2014), implying a long-term mechanism underlying the evolution of recombination frequency (Stapley et al., 2017). For instance, the triploid hybrids of *Brassica* showed the highest recombination frequency in a comparative analysis with hybrids of diploid (AA), allotriploid (AAC), and allotetraploid (AACC) in the same genomic background (Leflon et al., 2010). Our previous research has proved that the hexaploidization of wheat could increase the recombination frequency of the D genome by more than twofold (Wan et al., 2021). In the current study, compared with the synthetic hexaploid populations, the mean recombination frequency between adjacent and linked SNP loci was significantly (*P* < 0.01) increased in the pentaploid hybridization-derived populations based on the genotyping data. Consequently, the genetic interval and full lengths of genetic maps were substantially increased in pentaploidization compared to hexaploidization. To directly and conveniently comparative analyze the variations of recombination frequency and genetic distance in recombination pentaploid and hexaploid wheat populations, the same polymorphic SNP markers were used in each recombination population set of SPW and SHW, and the genetic maps were constructed in line with the physical position of SNP markers (“By Anchor Order” and “Algorithm By Input” functions). Although the calculation approach might inflate the length of genetic maps due to ignorance on the location change of SNP markers, the current study paid much more attention to the variations of recombination frequency between two adjacent and linked SNP loci. Moreover, a similar increasing tendency of chromosome recombination has been identified in the comparison of pentaploid and hexaploid recombination populations through self-organized genetic maps using the nnTwoOpt algorithm method. In addition, there might be a genetic differentiation of the D genome of hexaploid wheat compared to the D genome of *Ae. tauschii* species after the long-term evolution process, resulting in the differences in the polymorphic ratio of SNP markers and recombination frequency between SPW1 (the F_2_ progeny of the Chuanmai42 × Langdon) and SPW2 (the F_2_ progeny of the LS783 × Yuanwang) populations. More works are needed to analyze the influence mechanisms.

The recombination frequency is mainly affected by changes in the initiation of chromosome pairing, responses to the molecular control of chromosome pairing, and delayed or accelerated progression through the meiotic cell cycle stages (Martinez-Perez et al., 2001; Pecinka et al., 2011; Pelé et al., 2017). Polyploids with odd-ploidy levels might lead to alien chromosomes remaining as univalents during homologous pairing in the meiotic metaphase (Padmanaban et al., 2017, 2018). As early as 1924, Kihara (1924) had already reported that the F_1_ pentaploid wheat hybrids had 14 bivalents of AB chromosomes and seven univalents of D chromosomes. The chromosome number of individuals since the F_2_ generation, however, varies from 28 to 42 due to the elimination or retention of D chromosomes (Jenkins and Thompson, 1930; Love, 1940; Kihara, 1982; Wang et al., 2005; Lanning et al., 2008; Martin et al., 2011). Increased genomic instability may compensatorily enhance the crossover frequency among unaffected bivalents (Parker, 1975; Tease and Jones, 1976; Birchler et al., 2001; Carlton et al., 2006; Chen, 2007; Leflon et al., 2010). For example, the unpaired chromosomes in allotriploid hybrids were indicated as a factor driving the increase in crossover frequency during meiosis (Leflon et al., 2010; Mason et al., 2014). In the present study, the inter-ploidy hybridization between hexaploid wheat and tetraploid wheat significantly increased the homologous recombination of AB subgenomes. The increased recombination in allopentaploids may help generate new genotypes and eliminate linkage drag when introducing superior alleles or traits, stabilize the chromosome complement and segregation during meiosis, and accelerate the fixation of high-fitness genotypes.

The formation of crossovers is controlled by complex biological processes (Heyer, 2004; Lynn et al., 2007). Besides, the distribution of crossovers is consistently restricted to subtelomeric and especially pericentromeric regions. Recent research revealed that the pericentromeric and interstitial regions preserved closed chromatin organization during recombination initiation and became unpacked only later (Lenykó-Thegze et al., 2021). Therefore, the favorable allele combinations within a considerable proportion of the genome were prevented while ensuring plant fertility. However, the pentaploid hybridization might increase the inter-genomic exchange in the poor recombination regions. Two chromosomes 7A and 3B which harbored the most polymorphic SNPs markers, also some of them located in the pericentromeric region, were chosen to construct a consensus map of the four populations (Supplementary Figure 2) visualizing the recombination frequency variation between neighboring markers. Current results showed a high increase in the pericentromeric region of 7A and 3B (pseudomolecule position of centromere located in 360.2 to 363.8 Mb and 345.8 to 349.0 Mb region, respectively). For example, the mean recombination frequency of the 7A chromosome increased by 3.77% in SPW1 and 12.55% in SPW2; the mean recombination frequency of the 3B chromosome increased by 9.56% in SPW1 and 6.21% in SPW2 (statistical in a window size of ± 50 Mb of the physical location of centromere). Increased recombination was also detected in other regions along the whole chromosomes 7A and 3B. Earlier research indicated that a quantitative trait locus (QTL) on 7AL explained 18% of the variation in the number of spikelets (Kuzay et al., 2019). About 70% of the genes on chromosome 3B, which are associated with newly identified QTLs for grain yield, nitrogen use efficiency, plant height, and ear emergence, were detected in crossover-poor regions (Choulet et al., 2014). The desirable genes might be utilized in the pentaploid-derived progenies.

### Pentaploids Enrich the Genetic Diversity of Bread Wheat for Re-Evolution and Breeding

Inter-ploidy hybridization between hexaploid and tetraploid wheat leads to complex responses, including chromosome rearrangements, gain or loss of chromosomal segments, gene activation and suppression, variations in the epigenome, and activation of transposons (Matsuoka, 2011; Padmanaban et al., 2017), in parallel with increasing chromosome recombination frequency. The pentaploid-derived progenies combined both genetic variabilities from hexaploid and tetraploid wheat, which have the great potential that carried numerous novel combinations of beneficial alleles and an abundance of genetic diversity. Therefore, the progenies could be a useful gene pool for breeding in future. Besides, the pentaploids could help overcome the genetic bottleneck of bread wheat resulting from allopolyploidy and modern breeding and accelerate its re-evolution.

However, the chromosomal imbalance, genome instability, and incompatibility, in turn, induce abnormal development, reduced fitness, and reproductive failure (Liu et al., 2009; Padmanaban et al., 2017). Distorted segregation is a common phenomenon in plants, in which a given genotype class deviates from the expected Mendelian proportion of individuals (Xu et al., 1997; Yang et al., 2020). The genetic basis of distorted segregation was suggested to be the abortion of male or female gametes, or the selective fertilization of particular gametic genotypes (Xu et al., 1997). Our previous research detected an increasing distorted segregation ratio after allohexaploid hybridization in comparison to homoploid hybridization of D genomes (Wan et al., 2021). The current study also detected different levels of distorted markers in the F_2_ pentaploid and hexaploid populations. The pentaploid-derived population all showed a high distorted segregation rate (80.55% in SPW1 and 63.83% in SPW2) while the hexaploid-derived population showed a very low distorted segregation rate (4.79% in SHW1 and 6.42% in SHW2). The high proportion of distorted segregation loci that skewed in favor of the female parent genotype/allele in pentaploidization might promote to generate functional gametes and increase seed set and germination of the offspring, leading to increased fitness, survival, and reproduction (Burgess and Husband, 2004; Hernández-Castellano et al., 2020).

Triticeae polyploids often undergo recurrent hybridizations with their parents (Fan et al., 2013; Sha et al., 2017). Following the initial allohexaploidization event of bread wheat, the genetic diversity of tetraploid species with the AB genome may naturally introgressed into bread wheat through the intermediates of pentaploid hybrids (Merchuk-Ovnat et al., 2016). For example, ancestral QTL alleles associated with high drought resistance and productivity from wild emmer wheat have been detected in modern wheat cultivars (Merchuk-Ovnat et al., 2016). The introgression increases the genetic diversity of bread wheat and facilitates its natural evolution.

In modern wheat breeding, the pentaploid-derived progenies could be used to introgressive useful agronomic traits into bread wheat cultivars, including high-yield potential and diseases resistance (leaf rust, stripe rust, powdery mildew) (Reader and Miller, 1991; Leonova et al., 2002; Mohler et al., 2013; Xu et al., 2013; Yaniv et al., 2015; Simmonds et al., 2016). Moreover, the continuous selfing may result in nascent elite recombination and chromosome substitution or addition lines of hexaploid or tetraploid wheat, possibly after six or more generations, through the gain or loss of D chromosomes (Padmanaban et al., 2018; Yang et al., 2021).

There are several potential barriers (e.g., poor seed set, low fertility, and low fitness) to the generation of pentaploid wheat hybrids, leaving little attention being paid to commercial breeding (Padmanaban et al., 2017). Regarding this problem, we conducted reciprocal crosses between several hexaploid and tetraploid wheat in field trials. The average seed setting rates were about 50% in reciprocal crosses. The germination rates were more than 52.35% while the hexaploid wheat (high-ploidy) was employed as the male parent. However, the crosses of tetraploid wheat × hexaploid wheat only showed an average 7.87 and 8.42% germination rate. This result was in agreement with the findings discussed by Granhall (1943) and Sharma and Gill (1983) that crosses using the higher ploidy level species, as the female generally leads to higher levels of seed germination and seedling establishment. This study further proved that the identified barriers can be overcome by employing the higher ploidy level material as the maternal parent. This phenomenon might be contributed by the AABB component of hexaploid wheat which possesses a significantly stronger capacity to buffer and sustain imbalanced D genome chromosomes than the AABB genome of tetraploid wheat (Deng et al., 2018). Pentaploid introgression-based breeding depends on extensive recombination using hexaploid wheat as the maternal donor and tetraploid wheat as the minor parent.

In conclusion, the increased recombination of AB chromosomes in pentaploids, especially near the centromere region, will lead to abundant genetic variations. This, together with the prediction that the deleterious alleles of the minor parent may be removed more completely from genomic regions with a low recombination frequency (Wu, 2001), suggest that pentaploid introgressions are useful for wheat breeders who are interested in generating abundant genetic variations, eliminating linkage drag and deleterious genotypes, and breaking the genetic bottleneck of contemporary wheat.

## Data Availability Statement

The datasets presented in this study can be found in online repositories. The names of the repository/repositories and accession number(s) can be found in the article Supplementary Material.

## Author Contributions

WY, XF, and YZ conceptualized the study and contributed to the funding acquisition. FY, HW, and XF contributed to the methodology, formal analysis, and data curation. JL and ZL contributed to the project administration and resources. XZ, NY, QW, and YY contributed to the investigation. FY and XF wrote the original draft. XF, WM, and WY reviewed and edited the manuscript. All authors contributed to the article and approved the submitted version.

## Conflict of Interest

The authors declare that the research was conducted in the absence of any commercial or financial relationships that could be construed as a potential conflict of interest.

## Publisher’s Note

All claims expressed in this article are solely those of the authors and do not necessarily represent those of their affiliated organizations, or those of the publisher, the editors and the reviewers. Any product that may be evaluated in this article, or claim that may be made by its manufacturer, is not guaranteed or endorsed by the publisher.

## Supplementary Material

Supplementary Figure 1 Physical distribution of polymorphic markers in the AB genomes of two sets of pentaploid-hexaploid populations. The purple circle indicated the centromere location. **(A)** Populations SPW1 and SHW1. **(B)** Populations SPW2 and SHW2.

Supplementary Figure 2 Consensus map of chromosomes 7A and 3B in two sets of pentaploid and hexaploid populations. Genetic gaps are indicated in green. **(A)** Consensus map of chromosome 7A. **(B)** Consensus map of chromosome 3B.

Supplementary Data 1 Genotyping data of SPW1 population.

Supplementary Data 2 (Genotyping data of SHW1 population): Corrected the title AA-Chuanmai42 and A-Chuanmai42 to AA-Langdon/SQ665 and A-Langdon/SQ665, respectively; Corrected the title BB-Langdon and B-Langdon to BB-Chuanmai42 and B-Chuanmai42, respectively.

Supplementary Data 3 (Genotyping data of SPW2 population): Corrected the title AA-Chuanmai42 and A-Chuanmai42 to AA-Langdon/SQ783 and A-Langdon/SQ783, respectively; Corrected the title BB-Langdon and B-Langdon to BB-Yuanwang and B-Yuanwang, respectively.

Supplementary Data 4 (Genotyping data of SHW2 population): Corrected the title AA-Chuanmai42 and A-Chuanmai42 to AA-Langdon/SQ783 and A-Langdon/SQ783, respectively; Corrected the title BB-Langdon and B-Langdon to BB-Yuanwang/SQ783 and B-Yuanwang/SQ783, respectively.
