# Supplementary figures and images for: Pentaploidization Enriches the Genetic Diversity of Wheat by Enhancing the Recombination of AB Genomes
Source: Front Plant Sci. 2022 Jun 29;13:883868. doi: 10.3389/fpls.2022.883868 (PMC9281561; doi:10.3389/fpls.2022.883868)

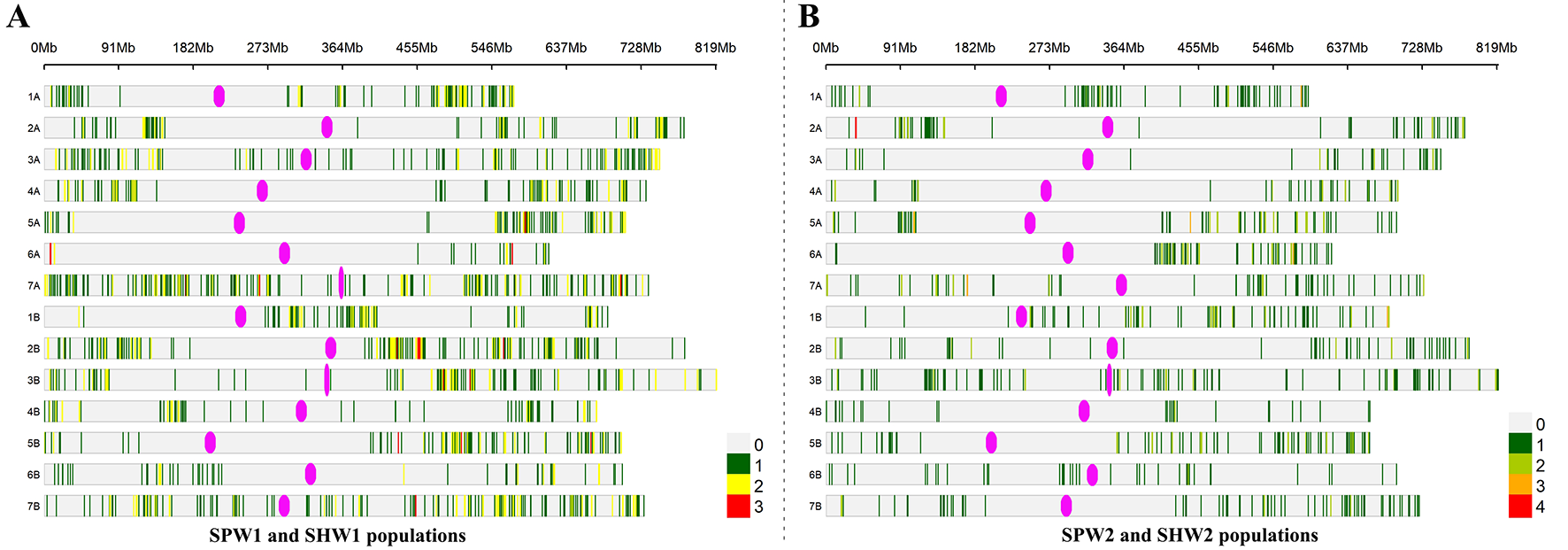

Supplement: Supplementary Figure 1 — Physical distribution of polymorphic markers in the AB genomes of two sets of pentaploid-hexaploid populations. The purple circle indicated the centromere location. (A) Populations SPW1 and SHW1. (B) Populations SPW2 and SHW2. [file Image_1.TIF]

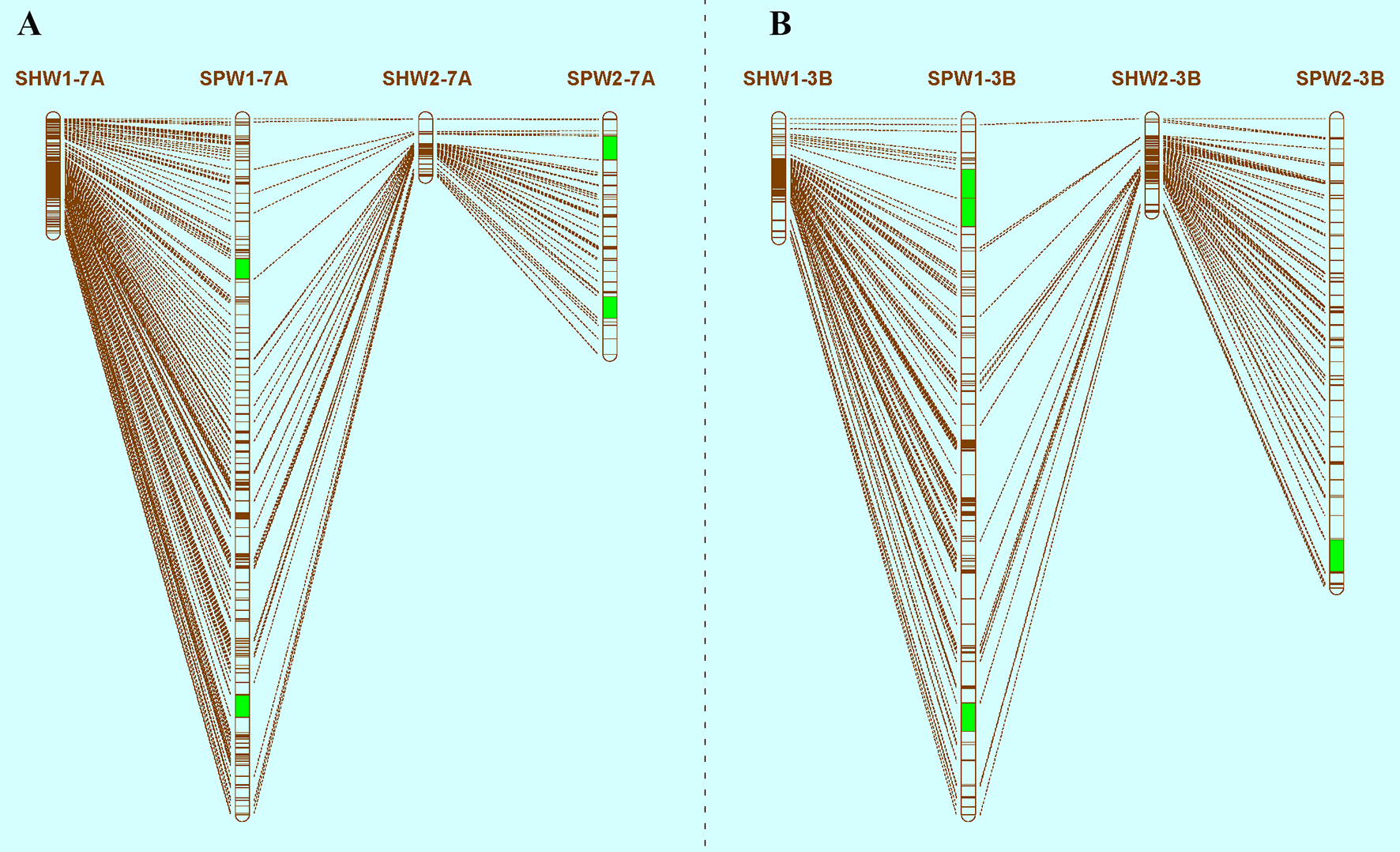

Supplement: Supplementary Figure 2 — Consensus map of chromosomes 7A and 3B in two sets of pentaploid and hexaploid populations. Genetic gaps are indicated in green. (A) Consensus map of chromosome 7A. (B) Consensus map of chromosome 3B. [file Image_2.TIF]
